# Effects of vitamin D supplementation on cardiovascular risk factors in shift workers: Study protocol for randomized, double-blind, placebo-controlled clinical trial

**DOI:** 10.1097/MD.0000000000015417

**Published:** 2019-05-03

**Authors:** Virginia Capistrano Fajardo, Fernando Luiz Pereira de Oliveira, George Luiz Lins Machado-Coelho, Fausto Aloísio Pedrosa Pimenta, Silvia Nascimento de Freitas, Antônio Luiz Pinho Ribeiro, Maria Marta Sarquis Soares, Marcio Weissheimer Lauria, Rosielle da Costa Farias, Ive Bahia França, Raimundo Marques do Nascimento Neto

**Affiliations:** a Applied Science to Adult Health Postgraduate Program, School of Medicine, Federal University of Minas Gerais, Belo Horizonte; b Department of Statistics, Institute of Exact and Biological Sciences; c Department of Family Medicine, Mental and Collective Health, School of Medicine; d Department of Pediatric and Adult Clinics, School of Medicine; e Health and Nutrition Postgraduate Program, School of Nutrition, Federal University of Ouro Preto, Ouro Preto; f Department of Clinical Medicine, School of Medicine, Federal University of Minas Gerais, Belo Horizonte; g Applied Statistics and Biometrics Postgraduate Program, Department of Statistics, Federal University of Viçosa, Viçosa, MG; h School of Medicine, University Center of Espirito Santo. Colatina, ES, Brazil.

**Keywords:** cholecalciferol, cholesterol, hypertriglyceridemia, low-density lipoprotein, triglycerides, waist circumference

## Abstract

**Objective:**

The present clinical study aims to describe protocol to evaluate the effects of vitamin D3 supplementation on the cardiovascular risk factors in a population of rotating shift workers.

**Design:**

A randomized, double-blind, placebo-controlled, parallel group clinical trial testing 2 oral dosages of cholecalciferol (14,000 IU and 28,000 IU per week) for 12 months.

**Setting:**

The primary outcome for evaluation is an 18% reduction in hypertriglyceridemia (≥150 mg/dL) between pre and postintervention measurements. Baseline characteristics of the study population will be summarized separately within each randomized group, and will use tests for continuous and categorical variables. For all tests, a *P* < .05 will be considered significant. The analysis of primary and secondary outcomes will use an intention-to-treat population and a per-protocol population. The primary and secondary outcomes will be compared separately between each treatment group and placebo, using binary logistic regression or regressão de Poisson for proportions (for binary outcomes) and using linear regression for differences in means (for continuous endpoints), with 95% confidence intervals.

**Participants:**

Rotating shift workers, adults aged between 18 and 60 years, with hypovitaminosis D and alterations in at least 1 of the following parameters: fasting glucose, high-density lipoprotein cholesterol, triglycerides, low-density lipoprotein cholesterol, blood pressure, and waist circumference.

**Conclusion:**

This clinical trial aims to contribute to the gap in knowledge about the potential, dose, and time of vitamin D supplementation to generate beneficial effects on triglycerides in a population at increased risk for hypertriglyceridemia and vitamin D deficiency.

## 1 Introduction

Hypovitaminosis D has affected populations in different age groups around the world.^[1,2]^ In the United States, Canada, and Australia, mean serum levels of 20 to 30 ng/mL were found,^[2]^ and the prevalence of hypovitaminosis D (<30 ng/mL) in Brazil ranged from 5.7% to 52.9% in men older than 18 years.^[1]^ There are still controversies about the reference values of vitamin D [25(OH)D]; the Brazilian Society of Endocrinology and Metabolism^[1]^ and the Endocrine Society^[3]^ define sufficient levels as values ≥30 ng/mL, and the Institute of Medicine^[4]^ defines sufficient levels as values ≥20 ng/mL. The literature data on vitamin D status in occupational groups are still scarce. However, when analyzing vitamin D status according to occupational activity, it is observed that shift workers and night-shift workers have lower levels of vitamin D and higher prevalence of deficiency and insufficiency when compared to day workers.^[5,6]^

Shift workers have a higher prevalence of risk factors for cardiovascular diseases, such as increased glucose, increased blood pressure, altered lipid profiles, and obesity when compared to day-shift workers.^[7–9]^ Shift work leads to deregulation of the circadian cycle, which promotes metabolic changes such as deregulation of cortisol, leptin, ghrelin, and melatonin, which contribute to the development of these risk factors.^[7,10]^ In a previous study by our research group with rotating shift workers, Batista et al^[11]^ showed that workers with hypovitaminosis D (<30 ng/mL) had a 5.9 times greater chance of having altered low-density lipoprotein (LDL) cholesterol (≥160 mg/dL) and a 2.3 times greater chance of having altered triglycerides (≥150 mg/dL) in relation to workers with sufficient levels of vitamin D.

Cross-sectional studies of several age groups suggest that hypovitaminosis D is associated with cardiovascular disease and risk factors, isolated or clustered, such as metabolic syndrome, waist circumference, body mass index (BMI), fasting glucose, blood pressure, total cholesterol, high-density lipoprotein (HDL) cholesterol, LDL cholesterol, and triglycerides.^[12–16]^ It is not yet clear whether there is a cause–effect relationship. There are few randomized clinical trials and with inconsistent results to provide an evidence on the benefit of vitamin D in reducing the risk of extraskeletal diseases, and most of those studies have been done in postmenopausal women^[17,18]^ (above 50 years), with normal sleep–wake rhythms. Thus, there are no data in the literature on the effects of vitamin D replacement in adult men (<60 years) who work in shifts.

Considering that there is still no consensus on the dose and time required for vitamin D replacement to generate beneficial effects in the cardiovascular profile, this clinical study aims to describe protocol to evaluate the effects of vitamin D3 supplementation at doses of 14,000 IU and 28,000 IU weekly for 12 months on the cardiovascular risk factors in a population of rotating shift workers. The hypothesis of the present study is that there will be an 18% reduction in hypertriglyceridemia (≥150 mg/dL) between pre and postintervention measurements in adult males with work activity that breaks the usual circadian cycle.

## 2 Methods

### 2.1 Trial design

A randomized, double-blind, placebo-controlled, parallel group clinical trial testing 2 oral dosages of cholecalciferol (14,000 IU and 28,000 IU) per week for 12 months with a 1:1:1 allocation ratio. This study will demonstrate the efficacy of supplementation with both doses of vitamin D in reducing hypertriglyceridemia.

### 2.2 Participants

The study participants consisted of rotating shift workers from 5 units of a Brazilian iron ore mining company; all were adults aged between 18 and 60 years. The company's rotating shift schedule is 6 hours of work, followed by 12 hours of rest. All participants work 4 shifts on the following schedule: 7 PM to 1 AM, 1 PM to 7 PM, 7 AM to 1 PM, and 1 AM to 7 AM. After completing the 4-shift weekly cycle, they have a day off. The interviews and blood sample collection will occur in the workplaces.

The inclusion criteria are as follows:

- 25(OH)D levels <30 ng/mL (75 nmol/L)^[1]^
  With at least 1 of these parameters:
- Fasting glucose ≥100 mg/dL (5.55 mmol/L)^[19]^; HDL cholesterol <50 mg/dL (1.29 mmol/L) for women and <40 mg/dL (1.04 mmol/L) for men^[19]^;
- Triglycerides ≥150 mg/dL (3.88 mmol/L)^[19]^;
- LDL cholesterol ≥130 mg/dL (3.36 mmol/L)^[20]^;
- Blood pressure systolic ≥130 mm Hg or diastolic ≥85 mm Hg^[19]^;
- Waist circumference ≥80 cm for women and ≥90 cm for men.^[19]^

Subjects are excluded with evidence as following: malabsorption syndrome, kidney, liver, or thyroid diseases; specific drug use (anticonvulsants, corticosteroids, hormones, or vitamin and mineral supplements); or altered levels of serum creatinine, parathyroid hormone (PTH), serum calcium, or albumin. Among women, current breast feeding or pregnancy are also considered exclusion criteria.

After each visit (every 4 months), the follow-up will be interrupted if subjects evidence 25(OH)D >50 ng/mL (or 159 nmol/L) associated with altered serum calcium levels (>11 mg/dL) and with confirmation of a second test with altered serum calcium (>11 mg/dL) and PTH (<10 pg/mL) levels or evidence of vitamin D intoxication symptoms (dizziness, headache, nausea, rash, urticaria, weakness, numbness, and constipation); recent diagnosis of malabsorption syndrome, kidney, liver, or thyroid diseases; specific drug use (anticonvulsants, corticosteroids, hormones, or vitamin and mineral supplements); or participant request. In cases of interrupted due to intoxication, the code will be broken to clarify the diagnosis, only by the code keeper.

### 2.3 Interventions

This trial use 2 interventions groups and a placebo group. One of the intervention groups will receive 14,000 IU of cholecalciferol oral supplementation per week, and the other intervention group will receive 28,000 IU of cholecalciferol oral supplementation per week. The active compound is cholecalciferol (vitamin D3) from a pill containing 7000 IU per unit (SanyD) already existing in the Brazilian market (Aché Laboratórios Farmacêuticos S.A., São Paulo, Brazil).

For the double-blind masking of the groups, the vitamins were distributed as capsules; the encapsulation process was carried out by Amphora specialized handling pharmacy. Inside each capsule, the pills were intact to maintain the phycochemical integrity of the active compound. The placebo group's capsules contained only the inert formulation with the same color, smell, and weight of the other capsules distributed to the intervention groups.

Participants were individually instructed, by the responsible team, to take 1 capsule per week, preferably close to the time of eating large meals (lunch/dinner) or foods containing fat. The delivery of the capsules was carried out every 2 months with support from the company's medical team to accommodate the varied to work shifts and workplaces. Each participant has received a bottle, identified by the name of the participant, containing capsules for 10 weeks at each follow-up visit, with the last bottle containing capsules for 12 weeks, completing 52 weeks of intervention.

Compliance is ascertained by interview at follow-up visits and by phone, or participants’ message to the responsible team. To improve adherence, the responsible teams send a message every week for participants to remember to take the pill, also send messages with information about healthy lifestyle, and deliver the lipids tests results to participants after follow-up visits every 4 months.

### 2.4 Outcome measures

The primary outcome to be evaluated is an 18% reduction in hypertriglyceridemia (≥150 mg/dL) between the pre and postintervention measurements. The secondary outcomes to be evaluated are a 36% reduction in hypertriglyceridemia (≥150 mg/dL) between the pre and postintervention measurements with 28,000 IU of vitamin D; an 18% reduction in high LDL cholesterol (≥130 mg/dL) between the pre and postintervention measurements; an 18% reduction in waist circumference (≥90 cm men; ≥80 cm women) between the pre and postintervention measurements; and a reduction in low cognitive impairment (by cognition tests) between the pre and postintervention measurements.

### 2.5 Safety

The 25(OH)D used in this trial already exists in the market, which has safety and quality. It was decided to 2 doses of vitamin D: 14,000 IU and 28,000 IU per week. The first one corresponds to maintenance therapy (1500 to 2000 IU per day) for replacement adult population at risk for hypovitaminosis D, in accordance with the Brazilian Society of Endocrinology and Metabolism^[1]^ and Endocrine Society.^[3]^ The second is double of maintenance therapy. It was decided to use this low dose to verify if the maintenance therapy is able to reduce hypertriglyceridemia, and double dose in order to increase the probability of generating a significant difference in the vitamin D status between the treatment groups and placebo. The clinical trial lasts for 12 months due to the seasonality of the incidence of ultraviolet radiation.^[21]^ In addition, vitamin D has endogenous synthesis via exposure to the sun and can be stored in adipose tissue,^[3]^ factors that make the placebo group necessary.

The risk of intoxication should be considered, but this condition is rare and caused by the intake of extremely high doses of vitamin D for long periods. The clinical symptoms of intoxication are characterized by hypercalcemia, hyperphosphatemia, suppression of PTH levels which can lead to nephrocalcinosis, and soft tissues calcification, especially blood vessels, in addition to fatigue, weight loss, anorexia, and impairment of renal function.^[1,3]^ The safe value of serum vitamin D levels suggested to avoid hypercalcemia is less than 150 ng/mL.^[3]^

Safety evidence for 25(OH)D supplementation has been reported by the Endocrine Society^[3]^ and by others authors, who did not observe serious adverse events. In a randomized trial, vitamin D supplementation with 6000 IU daily for 3 months, after 3000 IU daily for more 3 months and 2200 IU daily for more 3 months, was not associated with any adverse events or hypercalcemia during all 9 months of the study.^[22]^ In the vitamin D, diet and activity study, 2000 IU of cholecalciferol daily were supplemented for 12 months, and 4 women reported light-headedness, severe headaches, nausea, rash/hives, weakness/numbness, and constipation.^[17]^

Symptoms of vitamin D intoxication include dizziness, headache, nausea, rash, urticaria, weakness, numbness and constipation, hypercalcemia, hypercalciuria, and associated kidney stones. To minimize the risk of vitamin D supplementation, individuals with a diagnosis of renal, hepatic, or thyroid disease were excluded. As a safety precaution, participants with 25(OH)D >50 ng/mL (or 159 nmol/L) associated with altered serum calcium levels (>11 mg/dL) and confirmation of a second test with altered serum calcium (>11 mg/dL) and PTH (<10 pg/mL) levels or evidence of vitamin D intoxication symptoms (dizziness, headache, nausea, rash, urticaria, weakness, numbness, and constipation) are excluded from trial. Additionally, measures will be taken to record adverse events or reactions: by the follow-up questionnaire every 4 months or in spontaneously by the participant by telephone/message or email to the responsible team.

### 2.6 Sample size

The sample was calculated with the following assumptions: the percentage of hypertriglyceridemia in the group not exposed to vitamin D replacement (40%)^[23]^; the percentage of hypertriglyceridemia in the group exposed to vitamin D replacement (22%)^[11]^; and the likelihood ratio equal to 2.3^[11]^, considering 80% power and a 95% confidence level. As a result, this study requires 106 participants in each group; after adding 10% for losses, a total of 350 participants at least were enrolled. To estimate the sample size, OpenEpi software (Open Source Epidemiologic Statistics for Public Health, EUA) version 3.01, a web-based epidemiologic and statistical calculator for public health, was used.

### 2.7 Recruitment

All rotating shift workers from 5 units of an iron ore mining company are invited by study researcher to participate in screening for a general health assessment. The screening visit is used to identify eligible participants. After identifying the potential participant, the study investigator invites the individual to participate in the clinical trial. The invitation is made for individual or group face-to-face interviews, to clarify any doubts, to sign the consent form and to delivery to the participant a copy of the consent form.

### 2.8 Randomization

Randomization will be used to ensure a balance between the 2 intervention groups and placebo group for deficient and insufficient vitamin D frequencies. The groups were also balanced by the following cardiovascular risk factors: fasting glucose, HDL cholesterol, triglycerides, LDL cholesterol, blood pressure, and waist circumference (Table 1).

**Table 1:**
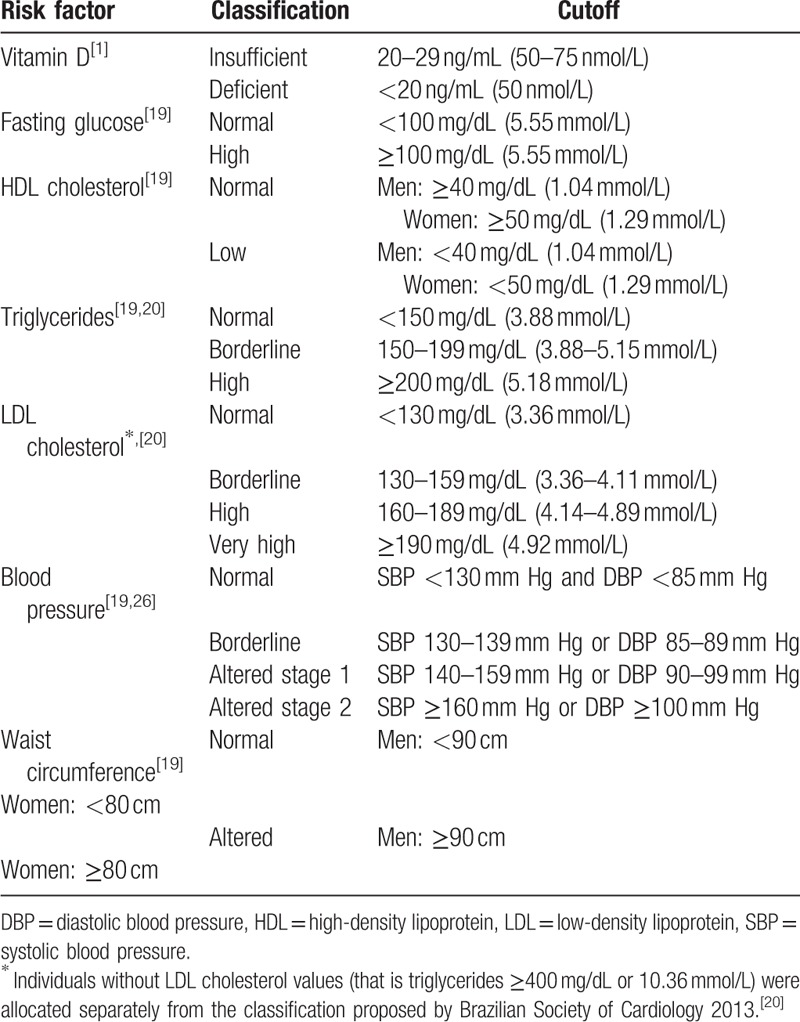
Criteria for allocating participants: parameters and their categories.

The allocation of the individuals were performed by an algorithm developed in R software version 3.5.1 [R Development Core Team (2018), R Foundation for Statistical Computing, Vienna, Austria] language that guarantees the similarity of the groups in relation to the total sample (eligible) considering a final sample (selected) weighted by the parameters and categories considered (Table 1). This method allocates the individuals with the greatest similarity between the frequencies of the categories of each parameter of the total sample among the subgroups of the clinical trial,^[24]^ similar to propensity score matching.^[25]^ The steps used in the allocation process of the patients in the groups of this clinical trial are presented below, considering a sample of *p* variables with a total of *m* categories:

First step: Initially, the algorithm performs the data reading, registers the number of categories present in each variable, and stores them in an ordered vector;

Second step: The algorithm receives the proportions of the categories of each variable and creates an ordered vector containing the proportions of the categories of each variable of the complete original sample *Pobs* *=* (*p*_1_, *p*_2_…, *p*_3_);

Third step: It randomly divides the initial sample into *k* groups of equal size *n*/*k*, where *n* is the sample size. If the result obtained from *n*/*k* is not an integer algorithm, the size of each group will be rounded to the next integer. Thus, the number of elements in the groups is defined;

Fourth step: After dividing the *k* groups, the proportions of the categories of each variable and each of the groups are calculated according to 1st and 2nd steps and the proportions vector *P*_1*k*_ = (*P*_2*k*_,*…, P*_*mk*_);

Fifth step: Sum of the Euclidean distances between the vectors of proportions of the *k* subsamples and the original proportions of the total sample, is defined as *D*_0_. That is: 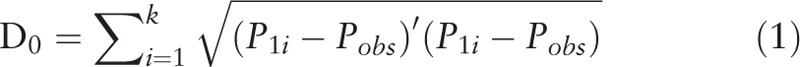


This will be the metric used in the simulation.

Sixth step: New subsamples are generated according to 4th step and a new metric is calculated for the new subsamples according to 6th step, namely: 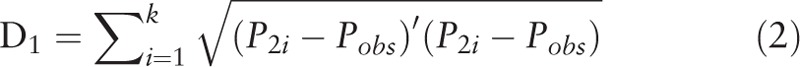


Seventh step: The subsample metrics are compared. The sample whose subgroup division presents the lowest value of *D* is maintained as the most probable sample to the original and will be the basis of the next simulation. The sample with the highest value of *D* will be discarded.

Eighth step: Repeat 6th–7th steps until the metric *D* reaches a predetermined stopping criterion or until a certain maximum number of simulations occurs. The sample with the lowest *D* value will be the one with the highest similarity in terms of the proportion of the categorical variables between the groups when compared to the original proportions.^[24]^

## 3 Blinding

Double-blind masking was maintained for the participants and the team members in direct contact with the participants. Only 1 researcher in the Laboratory of Cardiometabolism, School of Medicine of the Federal University of Ouro Preto, who is not involved in the data collection, has the knowledge of this information. This researcher was responsible for allocating the participants to the intervention or placebo groups and for receiving the packaged and encapsulated supplements.

### 3.1 Data collection

The screening was performed in a population of 686 rotating shift workers; all individuals were invited to a general health assessment. All data were collected at the company's clinic through face-to-face interviews with a structured questionnaire assessment and a blood draw. The team responsible was composed of nurses, biochemists, nutritionists, and students, who were trained to collect the data before the study.

**Figure 1:**
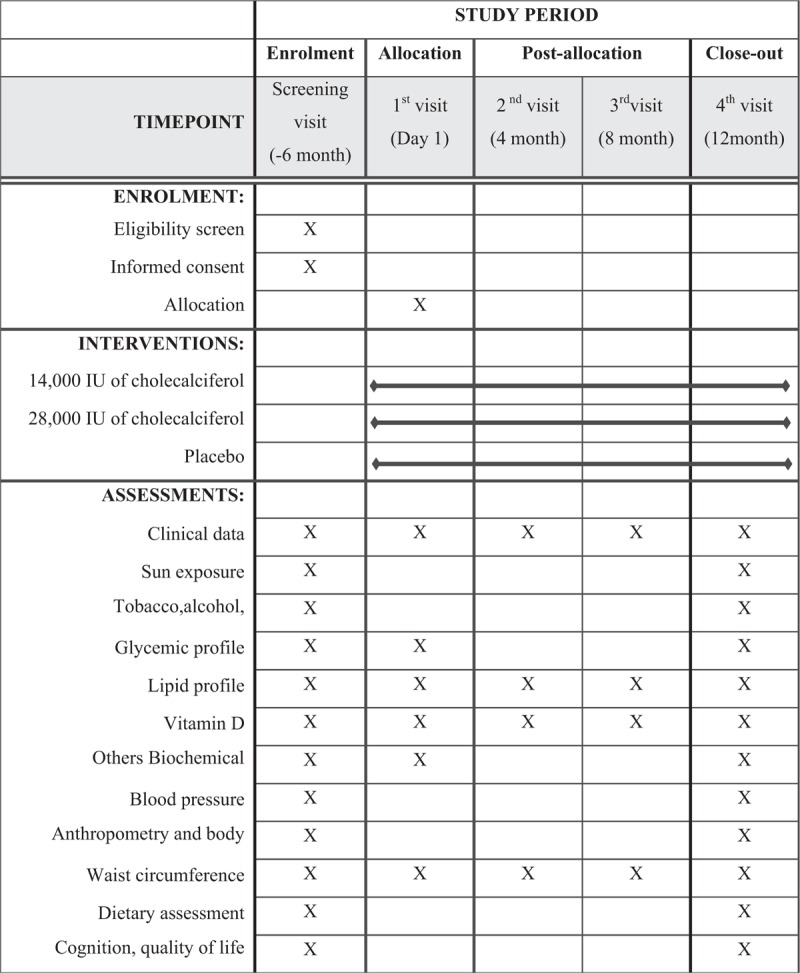
Clinical trial procedures schedule.

Figure 1 demonstrates all measurements that were evaluated at the screening visit, follow-up visits, and at the end of the 12-month supplementation period. At the screening visit, we are evaluating socioeconomic status, pre-existing diseases, drug use, sun exposure, lifestyle profile, quality of life, cognition (Table 2), blood pressure, biochemical data (Table 3), and anthropometrics and body composition measurements (Table 4). The follow-up visits will occur every 4 months and the supplementation delivery will occur every 2 months (it was integrated with follow-up visits). The follow-up visit procedures include blood sample collection, waist circumference measurement, and questionnaire interview. The vitamin D and lipid profile levels were quantified. The follow-up questionnaire monitors a presence of vitamin D intoxication, disease symptoms, drugs use, vitamins and minerals supplements, disease presence, treatment and others interventions during the trial, and food consumption alteration. At the end of the 12-month supplementation period, a reassessment of the general state of health was performed (Fig. 1).

**Table 2:**
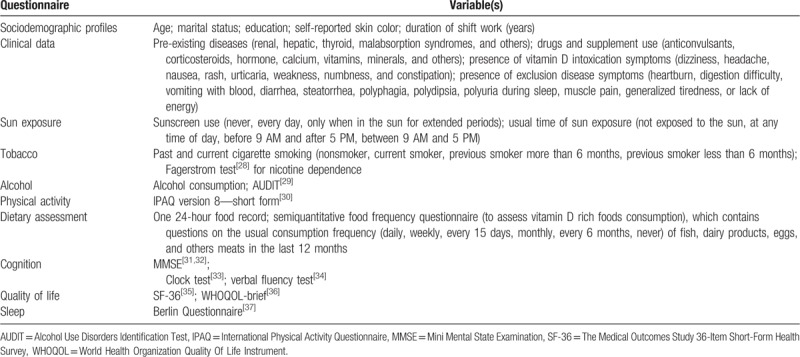
Components of interviews.

**Table 3:**
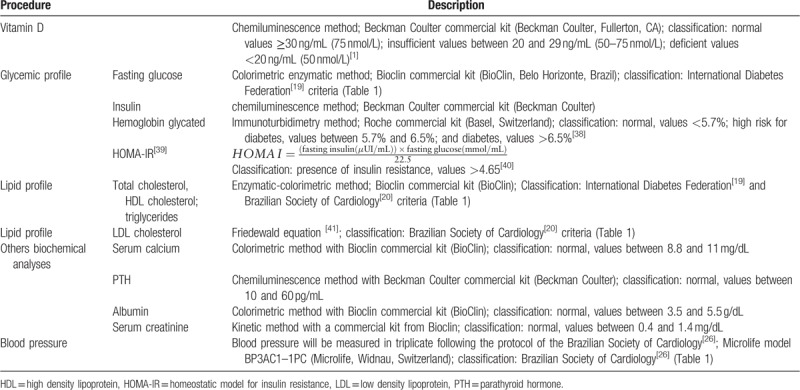
Biochemical analyses and blood pressure examinations and measurements.

**Table 4:**
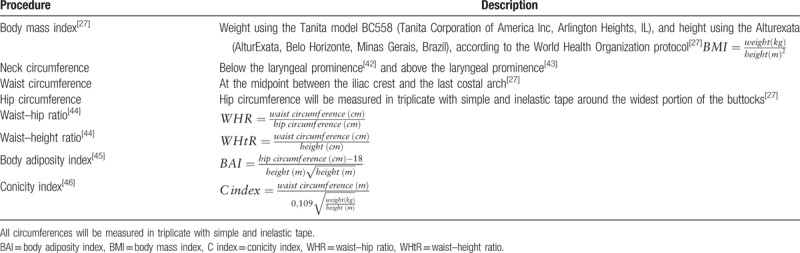
Anthropometry examinations and measurements.

The blood samples were collected after 10 hours of fasting by nurse and pharmacist of responsible team. Every follow-up visit the blood samples are analyzed immediately on fresh samples, and aliquots are stored frozen at −80°C. The procedures and biochemical analyses were performed by pharmacist of responsible team at the Laboratory of Pilot and Clinical Analyses (LAPAC/UFOP) and Laboratory of Epidemiology of Parasitic Diseases of the Federal University of Ouro Preto School of Medicine (Table 3).

Body composition was measured using the portable segmental tetrameric bioimpedance monitor Tanita model BC558 (Tanita Corporation of America Inc, Arlington Heights, IL). The following data were collected: body fat percentage (total and segmental), visceral body fat, skeletal muscle mass (total and segmental), total body water, total bone tissue, and basal metabolic rate. For the measuring process, the participant were standing barefoot in the platform center, without wearing or holding any heavy objects, looking straight ahead,^[27]^ with their feet placed on the 2 lower electrodes while holding the 2 upper electrodes with their arms extended away from the trunk, and without moving or talking during measurement. Four hours of fasting, an empty bladder, minimal dress, absence of heavy metal, and no strenuous exercise was also required before measurement, according to the Tanita manual (Table 4).

### 3.2 Statistical methods

Baseline characteristics and follow-up measurements of the study population will be summarized separately within each group. The baseline characteristics are vitamin D, fasting glucose, HDL cholesterol, triglycerides, LDL cholesterol, blood pressure systolic, waist circumference, cognition, sex, age, race, calcium, PTH, albumin, creatinine, total cholesterol, sun exposure, alcohol, physical activity, and food consumption. The follow-up measurements will be demonstrated as continuous variables, which are vitamin D, HDL cholesterol, triglycerides, LDL cholesterol, waist circumference, and total cholesterol. For continuous variables, the means and standard deviations or medians, 25th and 75th percentiles will be presented. The ANOVA or the Kruskal–Wallis test will be use for continuous variables after evaluating normality and the equality of variances using the Kolmogorov–Smirnov and Levene tests, respectively. For categorical variables, the number and percentage of participants within each category will be presented. The Pearson chi-squared test or Fisher exact test will be used. For each variable (continuous or categorical), the percentage of missing values will be reported. For all tests, a *P* < .05 will be considered significant.

The analysis of primary and secondary outcomes will use an intention-to-treat (ITT) population and a per-protocol (PP) population. ITT population includes all participants for whom outcome data are available, in the group to which they were randomized, regardless of the treatment actually received. PP population excludes individuals who did not comply with the protocol. The primary and secondary outcomes, which are hypertriglyceridemia, high LDL cholesterol, waist circumference, and low cognitive impairment, will be compared separately between each treatment group and placebo, using binary logistic regression or regressão de Poisson, if the outcome is rare, 5% or less, and the relative risk (RR) is less than about 2^[47]^ for proportions (for binary outcomes), and using linear regression for differences in means (for continuous endpoints), with 95% confidence interval (CI). An analysis will be performed to check whether adjusting for age, duration of shift work, physical activity level, alcohol consumption, lower cholesterol drugs, and foods consumption (fiber, total carbohydrates, total fat, and saturated fat) has any impact on the estimated treatment effects; if it has no impact, then they will not be included in the model. Statistical analyses will be calculated using PASW (Predictive Analytics Software, SPSS Inc, Chicago, IL) version 17.0 and R software (R Development Core Team) version 3.5.1.

### 3.3 Trial monitoring

This trial will be monitored by a data monitoring committee (DMC), Trial Steering Committee (TSC). That includes biostatistics, epidemiologist, cardiologist, occupational physician, nutritionist, and internal member of the company. The TSC performs the following indicators of the work execution, through reports and seminars. The DMC will perform the monitoring during and at the end of the study for endpoints settings and code opening. Interim analysis is performed to assess the sample losses after each follow-up visit.

### 3.4 Data management and quality assurance

A unique numeric identifier is assigned to each trial participant. All data are stored on the drive and in the cloud, and the links to this information are available only to the study coordination team. Consent forms and questionnaire data are stored in paper on Laboratory of Cardiometabolism of Medicine School of the Federal University of Ouro Preto. The questionnaire data are single-entered by the study team and the range checked twice for data values, to assure the quality of data entry.

### 3.5 Ethical considerations

This clinical trial was approved by the Research Ethics Committee of the Federal University of Ouro Preto (CEP 1.381.376) on December 17, 2015; and registered as “Fatigue Management Project” in the Brazilian Registry of Clinical Trials (RBR-6CS352). The responsible institution for this clinical trial is the Medicine School of Federal University of Ouro Preto. All participants sign consent form. If further other analyses will be required, prior authorization from the participants will be solicited.

If there is presence of side effects or proportion of individuals with hypervitaminosis higher than expected, there will be a change in protocol with communication to investigators, research ethics committee/institutional review board, trial participants, trial registries, journals, and regulators. This study does not have financial compensation, but to those who suffer damages resulting from the study participation, it is assured the referral to the company's health service.

The results of the trial will be reported to participants, health professionals and the public through seminars open to participants, technical report, and scientific articles.

## 4 Discussion

This is the first clinical trial evaluating 12 months of vitamin D supplementation in rotating shift workers. Shift work schedules are those that occur irregularly or uncommonly in comparison to usual daytime work (7/8 AM–5/6 PM), and can be fixed or rotating, continuous or discontinuous, and include or not include a night shift.^[48]^ Shift workers correspond to 14.8% of the workforce, with 2.5% rotating shift workers.^[49]^ In the mining area, the proportion of workers in rotating shifts is higher, reaching 31.9%.^[49]^

The shift work schedule allows activities 24 hours a day and aims to meet the needs of services, industries, and leisure.^[48]^ However, this work schedule contributes to the desynchronization of the circadian cycle. The circadian cycles are biological rhythms that are synchronized with external stimuli in order to adapt the organism to the environment in which it lives, thus allowing corporal homeostasis. Among the environmental factors, luminosity is considered the main external stimulus that promotes the synchronization of endogenous rhythms.^[10]^ Nighttime activities contribute to the misalignment of the circadian cycle, which can lead to adverse health consequences. Studies have reported that shift workers are at an increased risk for obesity, diabetes, metabolic syndrome, and cardiovascular diseases, as well as for increased glucose, triglycerides, total cholesterol, blood pressure, waist circumference and BMI, and for decreased HDL cholesterol ^[7–10,50]^.

In addition, Sowah et al,^[5]^ in a systematic review with 71 articles and a total sample of 53,345 workers, found, in shift workers, approximately 80% hypovitaminosis D (≤20 ng/mL), a higher risk of developing vitamin D deficiency (RR: 1.27; 95% CI: 1.26–1.28) and a higher risk of developing vitamin D insufficiency (RR: 1.24; 95% CI: 1.16–1.32) when compared to other occupational groups. This study suggests that these workers are more vulnerable to hypovitaminosis D. In a previous study conducted by our research group, with 391 rotating shift workers, we observed a high prevalence (73%) of hypovitaminosis D (<30 ng/mL).^[11]^

Therefore, the present study aims to contribute knowledge about the role of the dose and time of vitamin D supplementation in generating beneficial effects on triglycerides in a population at increased risk for hypertriglyceridemia and hypovitaminosis D.

### 4.1 Trial status

This trial is not concluded. This trial began with 362 participants enrolled in November 2016.

## Acknowledgments

Authors thank Medicine School and Nutrition School of Federal University of Ouro Preto for the transportation of interviews. Authors also thank Laboratory of Pilot and Clinical Analyses and Laboratory of Epidemiology of Parasitic Diseases of Medicine School of the Federal University of Ouro Preto for the equipment of procedures and biochemical analysis.

## Author contributions

**Conceptualization:** Fernando Luiz Pereira Oliveira, George Luiz Lins Machado-Coelho, Fausto Aloísio Pedrosa Pimenta, Silvia Nascimento Freitas, Raimundo Marques Nascimento Neto.

**Formal analysis:** Virginia Capistrano Fajardo, Fernando Luiz Pereira Oliveira.

**Funding acquisition:** Raimundo Marques Nascimento Neto.

**Methodology**: Virginia Capistrano Fajardo, Fernando Luiz Pereira Oliveira, George Luiz Lins Machado-Coelho, Fausto Aloísio Pedrosa Pimenta, Silvia Nascimento Freitas, Antônio Luiz Pinho Ribeiro, Maria Marta Sarquis Soares, Marcio Weissheimer Lauria, Rosielle da Costa Farias, Raimundo Marques Nascimento Neto.

**Project administration:** Fernando Luiz Pereira Oliveira, George Luiz Lins Machado-Coelho.

**Supervision:** Virginia Capistrano Fajardo, Fausto Aloísio Pedrosa Pimenta, Silvia Nascimento Freitas, Raimundo Marques Nascimento Neto.

**Writing – original draft:** Virginia Capistrano Fajardo.

**Writing – review & editing:** Fernando Luiz Pereira Oliveira, George Luiz Lins Machado-Coelho, Fausto Aloísio Pedrosa Pimenta, Silvia Nascimento Freitas, Antônio Luiz Pinho Ribeiro, Maria Marta Sarquis Soares, Marcio Weissheimer Lauria, Ive Bahia França, Raimundo Marques Nascimento Neto.
